# Temporal trends in mortality of tuberculosis attributable to high fasting plasma glucose in China from 1990 to 2019: a joinpoint regression and age-period-cohort analysis

**DOI:** 10.3389/fpubh.2023.1225931

**Published:** 2023-07-27

**Authors:** Chao Wang, Xueli Yang, Honglu Zhang, Yanzhuo Zhang, Jianfeng Tao, Xu Jiang, Chengai Wu

**Affiliations:** ^1^National Center for Orthopaedics, Beijing Research Institute of Traumatology and Orthopaedics, Beijing Jishuitan Hospital, Capital Medical University, Beijing, China; ^2^Department of Occupational and Environmental Health, School of Public Health, Tianjin Medical University, Tianjin, China; ^3^Department of Orthopaedics, Beijing Jishuitan Hospital, Capital Medical University, Beijing, China

**Keywords:** tuberculosis, Global Burden of Disease, high fasting plasma glucose, mortality, joinpoint regression, age-period-cohort analysis

## Abstract

**Background:**

Nowadays, high fasting plasma glucose (HFPG) has been identified as the important risk factor contributing to the increased burden of diseases. But there remains a lack of research on tuberculosis (TB) mortality specifically attributable to HFPG. Thus, this study aims to explore the long-term trends in HFPG-related TB mortality in China from 1990 to 2019.

**Methods:**

Data on HFPG-related TB mortality were obtained from the Global Burden of Disease (GBD) Study 2019. Analyzing the data using joinpoint regression and age-period-cohort methods adjusting for age, period, and cohort allowed us to assess the trends in TB mortality due to HFPG.

**Results:**

The age-standardized mortality rates (ASMRs) of TB attributable to HFPG exhibited a downward trend in China from 1990 to 2019, with an average annual percentage change (AAPC) of −7.0 (95% CI, −7.5 to −6.6). Similar trends were found for male (AAPC of −6.5 [95% CI, −7.0 to −6.0]) and female (AAPC of −8.2 [95% CI, −8.5 to −7.9]), respectively. Local drifts curve with a U-shaped pattern reflected the AAPC of TB mortality due to HFPG across age groups. The greatest decline was observed in the age group of 60–64 years. The mortality rates related to HFPG first increased and then decreased with increasing age, peaking in the 55–59 age group. Our analysis of the period and cohort effects found that the rate ratios of TB mortality due to HFPG have decreased over the past three decades, more prominently in women. It is noteworthy that while both genders have seen a decline in HFPG-attributable TB mortality and risk, men have a higher risk and slightly less significant decline than women.

**Conclusion:**

The present study shows that HFPG–related ASMRs and risk of TB in China decreased over the last 30 years, with similar trends observed in both men and women. In order to attain the recommended level set by the WHO, the effective strategies for glycemic control and management still needed to be implemented strictly to further decrease the burden of TB.

## 1. Introduction

Tuberculosis (TB) is a disease caused by an infection with *Mycobacterium tuberculosis* (MTB). Although the lungs are commonly affected by these bacteria, they can also invade other parts of the body, such as the bones, kidney and nervous system (1). Treatment for TB disease typically involves the administration of antibiotics, as the condition can be life-threatening if left untreated (2). TB still severely threatens the health of the world population (3). In China, the prevalence, incidence and mortality of TB have significantly reduced over the last 30 years. Based on the data obtained from the Global Burden of Disease Study 2017 (GBD 2017), a decline was observed in the prevalence (average annual percent change, AAPC: −0.5, 95% CI: −0.6% to −0.5%), incidence (AAPC: −3.2, 95% CI: −3.5% to −2.9%), and mortality (AAPC: −5.7, 95% CI: −6.2% to −5.3%) of TB in China from 1990 to 2017 (1). Zuo et al. utilized the Chinese Center for Disease Control and Prevention’s notifiable infectious disease reporting system to investigate the spatial–temporal characteristics and epidemiology of TB in China between 2004 and 2017. TB incidence decreased by 19.4% from 74.58/100,000 cases in 2004 to 60.08/100,000 cases in 2017. The AAPC was −3.3%, exceeded the global average of 2% (4). Nonetheless, TB remains an important issue of public health. China accounted for 8.4% of the total global incidence of TB, with approximately 840,000 new cases reported in 2019, placing it third among the thirty countries with the highest burden of this disease (5). With roughly 350 million individuals at risk for active TB disease, China bears the greatest global burden of latent TB infection (LTBI) (6). Moreover, both diabetes and hyperglycemia frequently lead to mortality and disability, either due to direct clinical consequences or an increased susceptibility to various diseases, including cardiovascular diseases, neurological disorders, respiratory infections, and TB (7–9). Due to population aging, increasing urbanization, and economic growth, China has witnessed one of the most dramatic rises in diabetes prevalence worldwide (10, 11). High fasting plasma glucose (HFPG) is a widely recognized indicator of hyperglycemia (12). In the GBD 2019 study, HFPG is characterized by fasting plasma glucose levels above 4.8–5.4 mmol/L, accounted for 11.3% of global all age-standardized deaths and 6.4% of disability-adjusted life-years (DALYs) across all causes (9). Previous evidence suggests that HFPG can contribute to an increased risk of several health outcomes, such as ischemic heart disease, stroke and chronic kidney disease (13). Furthermore, HFPG could increase risk of TB (14, 15).

TB patients who have diabetes or HFPG face a higher likelihood of experiencing more severe disease and unfavorable treatment outcomes when compared to TB patients without any co-morbidities (16, 17). For example, Boillat-Blanco et al. conducted a case–control study and found that TB patients with fasting hyperglycemia at enrolment had a higher risk of TB treatment failure or death (OR, 3.3; 95% CI, 1.2–9.3) (16). Specifically, long-term elevation in blood glucose levels can hinder the functioning of immune cells that play a crucial role in combating TB bacteria. Consequently, this weakened immune response provides an opportunity for TB bacteria to reproduce and spread more readily throughout the body, elevating the risk of developing active TB disease. Moreover, diabetes and hyperglycemia can hinder the body’s capacity to treat TB infections effectively (18–20). In comparison to other major risk factors like smoking and alcohol use, there appears to be a relatively lower level of disease awareness regarding HFPG. Smoking and alcohol use are typically associated with readily apparent and immediate health consequences, while HFPG is a condition characterized by elevated blood glucose levels that may not manifest noticeable symptoms in its early stages. As a result, individuals with HFPG may not experience immediate discomfort or obvious health problems, leading to limited awareness regarding the potential long-term health implications.

Thus, we used the data from the Global Burden of Disease Study 2019 (GBD 2019) to investigate and compare the temporal trends in TB-related mortality attributable to HFPG in China from 1990 to 2019. We employed joinpoint regression and age-period-cohort analysis for this purpose. The findings from this study could be valuable in guiding priority actions to prevent the TB burden attributable to HFPG and providing research-based evidence for managing HFPG in TB patients.

## 2. Methods

### 2.1. Overview and data sources

The GBD study which is a worldwide epidemiological undertaking sponsored by the Institute for Health Metrics and Evaluation, examines the impact of diseases and injuries in numerous countries across the globe. GBD 2019, the most recent version of the project, has amassed data on 369 diseases and injuries as well as 87 risk factors from the year 1990 to 2019 (21–23). The GBD research identifies three risk factors associated with TB, including HFPG (metabolic risk factor), smoking and drinking (behavioral risk factor). Moreover, the study offers precise explanations for these risk factors and outlines the TMREL. TMREL refers to the level of risk exposure that maintains the risk of disease at the population level to a minimum. HFPG is defined as any amount over the TMREL of 4.8–5.4 mmol/L (15, 21). We collected data on age-specific cases and mortality, and age-standardized mortality rates (ASMRs) for TB caused by HFPG in China from GBD 2019 for the period spanning 1990 to 2019 (24). The number of deaths was divided by corresponding mortality to obtain the population for each age group and year (25). Furthermore, the ASMRs should be determined using the global age distribution for 2019 as the standard population.

### 2.2. Statistical analysis

The analysis of temporal disease trends is an essential component of epidemiology that helps to inform the development of more precise prevention strategies, thereby enabling better control and management of diseases.

Firstly, we used the joinpoint regression model to examine the time trends in ASMRs of TB attributed to HFPG in China from 1990 to 2019. Long term trends of ASMRs were categorized into distinct segments and assessed the significance of the trends within every segment (25, 26). That is, the method could identify inflection points in the trend data and use them to divide the overall trend into smaller sub-segments, which evaluate the magnitude of the epidemiological trend (27). In the study, we could conduct an estimation of how the TB mortality attributable HFPG changes over time, and enable the identification of particular phases of growth or decline, provide valuable information on how the disease develops.

Then, we further conducted an age-period-cohort model to examine how a variable change over time, taking into account three temporal dimensions simultaneously. Specifically, the model estimated the effects of age, period, and birth cohort on the risk of HFPG-related TB mortality (28). The model distinguishes between age effects, which capture changes in the variable over an individual’s lifetime, period effects that represent environmental factors impacting the entire population, and birth cohort effects, which describe changes in the variable among people born in the same year who have experienced similar life events (29). The research investigated how HFPG affects TB mortality by analyzing various factors such as local drift, longitudinal age curve, period, and cohort rate ratios (RRs) (25). Specifically, the term local drift refers to the AAPC for each age group, taking into account the effects of period and birth cohort. Meanwhile, longitudinal age curve pertains to the age-specific rates that are adjusted for both cohort and period (25).

Moreover, in order to accurately analyze the effect of age, period, and cohort on the HFPG–attributable TB mortality, the data underwent a structuring process (30, 31). This involved organizing it into successive 5-year age groups (25–29, 30–34, …, 75–79), consecutive 5-year periods from 1990 to 2019 (1990–1994, 1995–1999, …, 2015–2019) and correspondingly generated consecutive birth cohorts. Notably, the period RRs or cohort RRs are adjusted for age and non-linear period or cohort effects in a given period or cohort when compared to a reference group (31–34). And we designated the reference groups as those comprising individuals aged 25–29 years, the period from 1990 to 1994, and belonging to the cohort born in 1965, respectively.

We conducted joinpoint regression analysis using joinpoint software version 4.8.0.1. The age-period-cohort analysis was conducted using the age-period-cohort web tool provided by the Biostatistics Branch, National Cancer Institute in Bethesda, MD. The tool can be accessed at https://analysistools.nci.nih.gov/apc/ (26, 27). Our statistical analyses were two-sided, and values with *p* < 0.05 were deemed statistically significant.

## 3. Results

### 3.1. Joinpoint regression analysis

**Figure 1:**
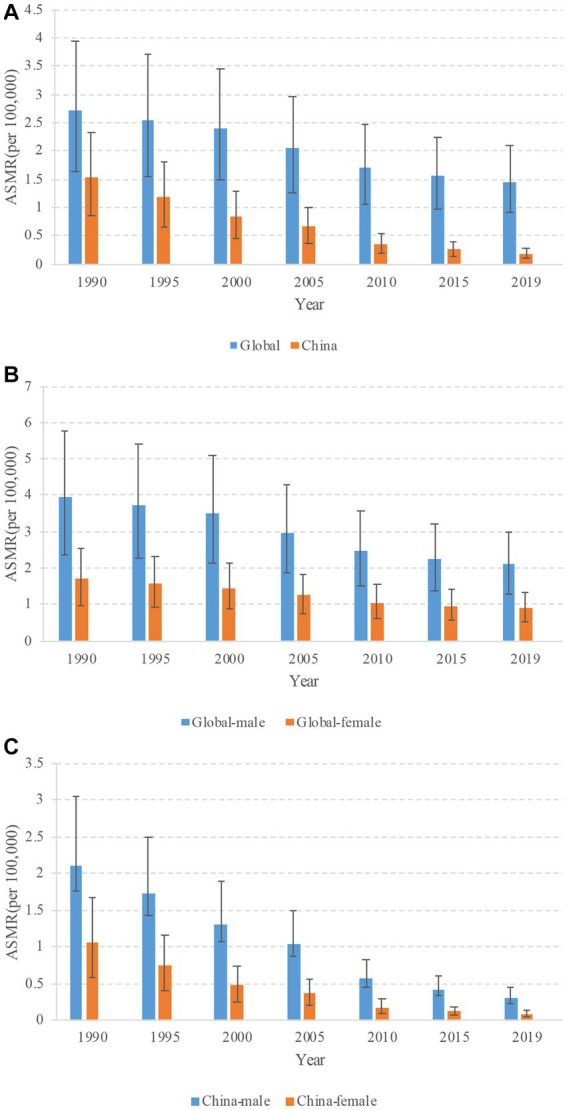
Trends in age-standardized mortality rates (ASMRs) of tuberculosis attributable to high fasting plasma glucose from 1990 to 2019. **(A)** Trends of ASMRs for both sexes in China and globally. **(B)** Trends of ASMRs for male and female in globally. **(C)** Trends of ASMRs for male and female in China.

**Figure 2:**
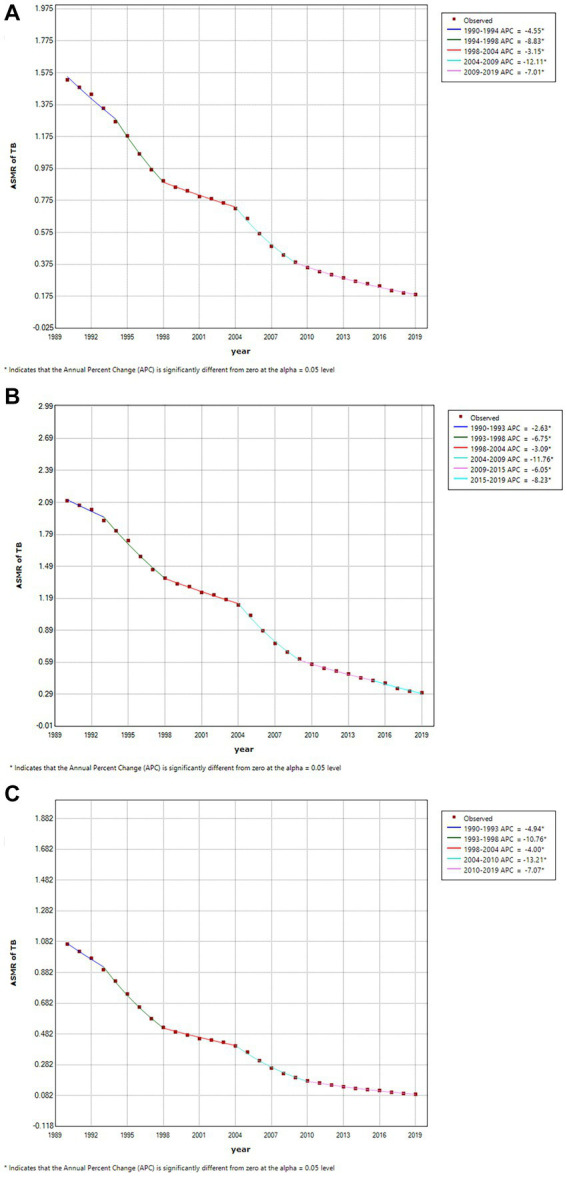
Joinpoint regression analysis in sex-specific age-standardized mortality rate (ASMR) of tuberculosis attributable to high fasting plasma glucose in China during1990–2019. **(A)** China-all. **(B)** China-male. **(C)** China-female. An asterisk indicates that the annual percent change is statistically significantly different from zero at the *α* = 0.05 level.

Globally, the trends in ASMRs of HFPG-related TB mortality demonstrated decreasing trends from 1990 to 2019, with higher TB mortality in male than in female. The ASMRs reduced from 2.7/100,000 in 1990 to 1.5/100,000 in 2019, from 3.9 to 2.1 for male, and from 1.7 to 0.9 for female, respectively (Supplementary Table S1). During the same period, the ASMRs decreased from 1.5/100,000 in 1990 to 0.2/100,000 in 2019 (AAPC of −7.0 [95% CI, −7.5 to −6.6]) in China. From 1990 to 2019, there was similar trends observed in the ASMRs for males (declining from 2.10 to 0.30 with an AAPC of −6.5 [95% CI, −7.0 to −6.0]) and females (decreasing from 1.1 to 0.10 with an AAPC of −8.2 [95% CI, −8.5 to −7.9]) (Table 1 and Figure 1). Furthermore, the findings indicate that the most significant decrease occurred during the years 2004–2009 for both male and the entire population, and 2004–2010 for female. Specifically, female experienced a more substantial decline than male, as illustrated in Figure 2.

**Table 1:** Sex- and age-specific high fasting plasma glucose-attributable tuberculosis mortality in China in 2019 and the average annual percentage changes (AAPC) from 1990 to 2019.

Country	Both		Male		Female	
	Rates in 2019, 95%UI (per 100,000 population)	AAPC, 95%CI (%, 1990–2019)	Rates in 2019, 95%UI (per 100,000 population)	AAPC, 95%CI (%, 1990–2019)	Rates in 2019, 95%UI (per 100,000 population)	AAPC, 95%CI (%, 1990–2019)
China						
ASMR	0.19 (0.11–0.29)	−7.0 (−7.5 to −6.6)	0.30 (0.17–0.48)	−6.5 (−7.0 to −6.0)	0.09 (0.05–0.15)	−8.2 (−8.5 to −7.9)
25–29 years	0.02 (0.01–0.03)	−5.6 (−7.2 to −4.1)	0.03 (0.01–0.04)	−5.0 (−6.1 to −4.0)	0.01 (0–0.01)	−7.1 (−9.1 to −5.1)
30–34 years	0.04 (0.02–0.06)	−6.0 (−7.1 to −4.9)	0.06 (0.03–0.09)	−5.3 (−6.7 to −3.9)	0.01 (0.01–0.02)	−8.0 (−9.8 to −6.2)
35–39 years	0.08 (0.04–0.12)	−6.1 (−6.6 to −5.6)	0.12 (0.07–0.19)	−5.3 (−5.7 to −5.0)	0.03 (0.01–0.05)	−8.2 (−8.9 to −7.4)
40–44 years	0.14 (0.08–0.21)	−6.3 (−6.8 to −5.9)	0.22 (0.12–0.34)	−5.7 (−6.1 to −5.3)	0.05 (0.03–0.09)	−8.2 (−8.9 to −7.5)
45–49 years	0.18 (0.11–0.28)	−7.1 (−7.8 to −6.3)	0.30 (0.17–0.47)	−6.5 (−7.1 to −5.9)	0.07 (0.03–0.11)	−8.6 (−9.2 to −8.0)
50–54 years	0.26 (0.15–0.38)	−7.7 (−8.1 to −7.2)	0.40 (0.24–0.62)	−7.1 (−7.8 to −6.4)	0.11 (0.06–0.18)	−9.1 (−9.5 to −8.6)
55–59 years	0.39 (0.23–0.59)	−7.8 (−8.3 to −7.2)	0.59 (0.35–0.91)	−7.3 (−8.0 to −6.7)	0.19 (0.11–0.30)	−8.8 (−9.5 to −8.0)
60–64 years	0.50 (0.27–0.77)	−7.9 (−8.5 to −7.4)	0.74 (0.39–1.15)	−7.5 (−8.1 to −6.8)	0.26 (0.14–0.44)	−8.8 (−9.4 to −8.2)
65–69 years	0.67 (0.23–1.17)	−7.6 (−8.2 to −7)	0.98 (0.34–1.78)	−7.3 (−8.0 to −6.6)	0.36 (0.12–0.68)	−8.4 (−9.1 to −7.6)
70–74 years	1.01 (0.24–1.96)	−7.1 (−7.6 to −6.6)	1.52 (0.33–2.96)	−6.7 (−7.4 to −6.1)	0.54 (0.13–1.09)	−8.1 (−8.3 to −7.8)
75–79 years	1.61 (0.55–2.89)	−6.6 (−7.1 to −6.2)	2.50 (0.82–4.59)	−6.3 (−6.7 to −5.8)	0.82 (0.27–1.49)	−7.7 (−8.1 to −7.3)

UI, uncertainty interval; CI, confidence interval; ASMR, age–standardized mortality rates; AAPC, average annual percent change.

### 3.2. Age-period-cohort analysis

#### 3.2.1. Variation with age

**Figure 3:**
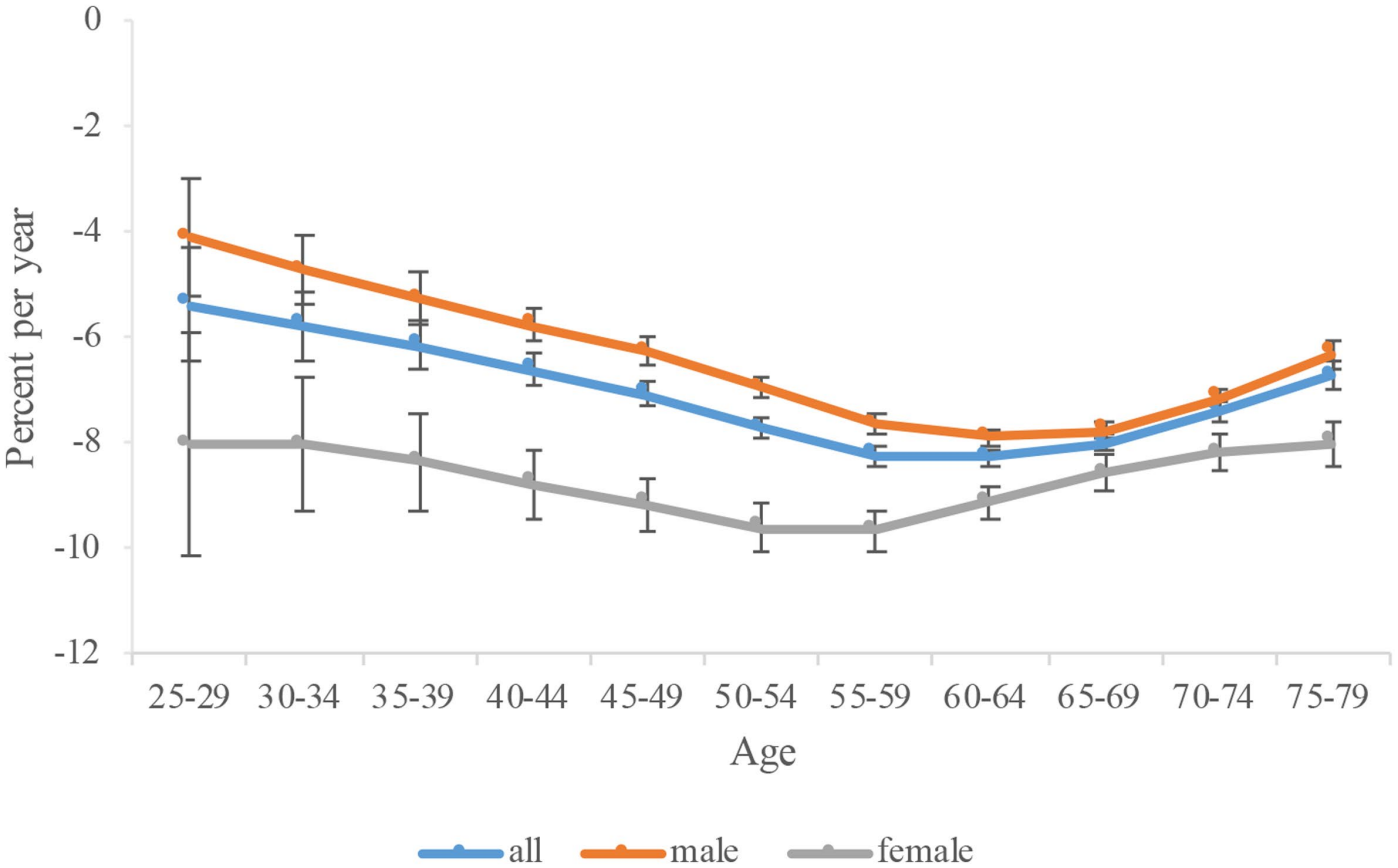
Local drift of high fasting plasma glucose-attributable tuberculosis mortality in China. The local drift value <0 revealed a decreasing trend, on the contrary, an increase ng trend. Error bars represent the 95% CIs for the local drift values.

To determine the age-specific effects of HFPG on TB mortality in China, we conducted an age-period-cohort analysis and calculated the AAPC. The U-shaped curve of local drifts for TB across age groups demonstrated the greatest decline among individuals aged 60–64 in whole population and men (Figure 3). Notably, women exhibited the most significant decrease in the 55–59 age group. Our results suggested that women experienced greater reductions than men across age groups.

**Figure 4:**
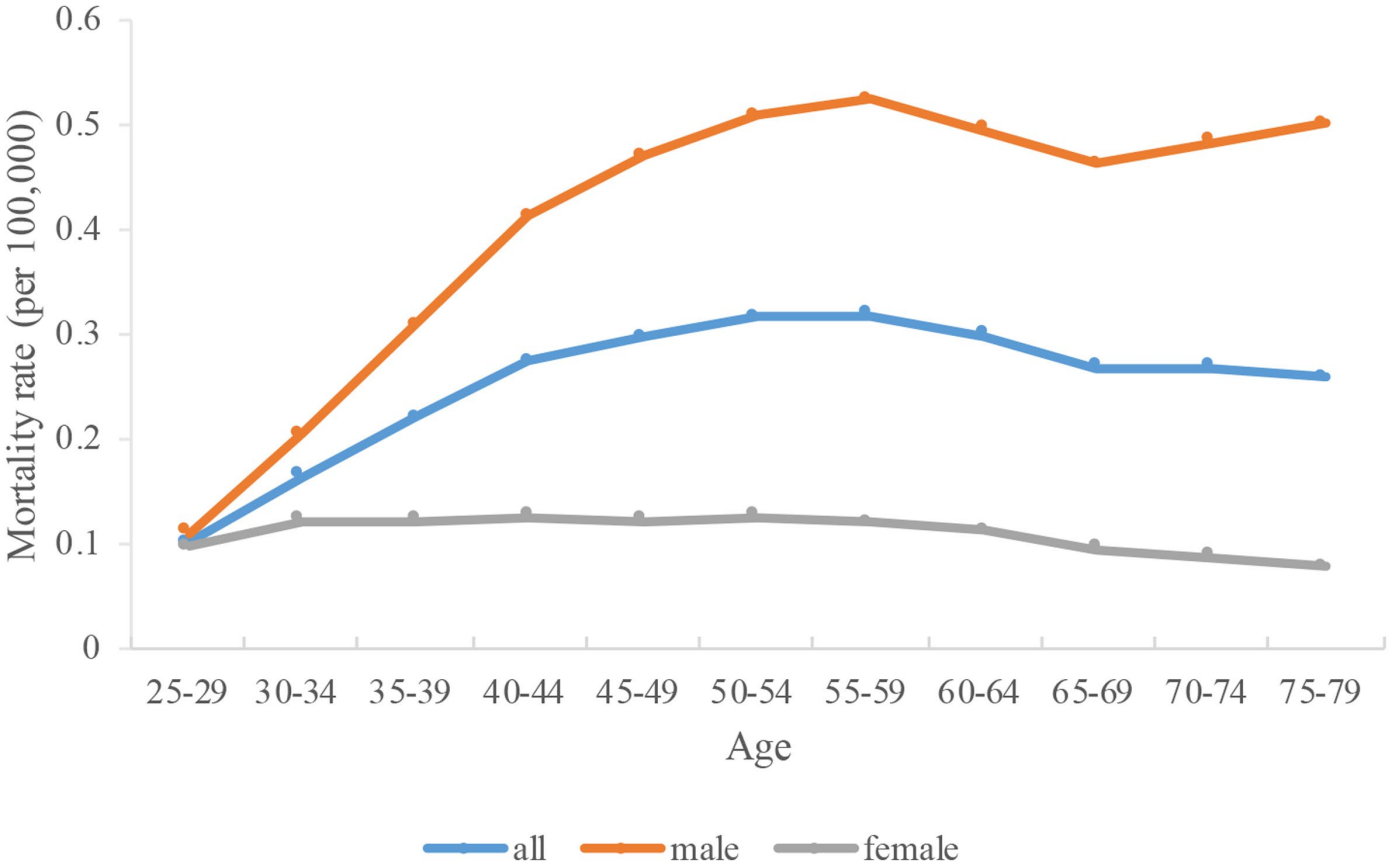
Longitudinal age curves of high fasting plasma glucose-attributable tuberculosis mortality in China. Data provided for all, male, and female. Fitted longitudinal age-specific rates of high fasting plasma glucose-attributable tuberculosis mortality (per 100,000).

Figure 4 shows the longitudinal age curves for HFPG-attributable TB mortality, which indicated that the mortality per 100,000 initially increases and then decreases with age. TB mortality increased from 0.1 in the 25–29 age group to 0.32 in the 55–59 age group (Table 2). The mortality peaks in the 55–59 age group and then begins to decline, and reach to 0.26 in age group 75–79. Regarding gender differences, the TB mortality in men increased from 0.11 in the 25–29 age group to 0.52 in the age group of 55–59 years. It also peaks in age group 55–59 and begins to decline, but rises from the 70–74 years and reached to 0.50 in age group 75–79. The TB mortality in women also first increased and then decreased with the increase of age. Nevertheless, it increased from 0.1 in the 25–29 age group to 0.13 in age group of 40–44 years. The mortality peaks in the 40–44 age group and then begins to decline, and reach to 0.08 in the 75–79 years old group. It’s important to highlight that male mortality were significantly higher than those of females across all age categories.

**Table 2:** Sex-specific high fasting plasma glucose-attributable tuberculosis mortality in China due to age effects.

Factor	Mortality in all, Rate (per 100,000)	Mortality in male, Rate (per 100,000)	Mortality in female, Rate (per 100,000)
Age			
25–29 years	0.10 (0.09–0.11)	0.11 (0.10–0.12)	0.10 (0.08–0.12)
30–34 years	0.16 (0.15–0.18)	0.20 (0.19–0.22)	0.12 (0.10–0.14)
35–39 years	0.22 (0.20–0.23)	0.31 (0.29–0.33)	0.12 (0.11–0.14)
40–44 years	0.27 (0.26–0.29)	0.41 (0.39–0.44)	0.13 (0.11–0.14)
45–49 years	0.30 (0.28–0.31)	0.47 (0.44–0.50)	0.12 (0.11–0.14)
50–54 years	0.31 (0.30–0.33)	0.51 (0.48–0.54)	0.12 (0.11–0.14)
55–59 years	0.32 (0.30–0.34)	0.52 (0.49–0.56)	0.12 (0.10–0.14)
60–64 years	0.30 (0.28–0.32)	0.49 (0.46–0.53)	0.11 (0.10–0.13)
65–69 years	0.27 (0.25–0.29)	0.46 (0.43–0.49)	0.09 (0.08–0.11)
70–74 years	0.27 (0.25–0.29)	0.48 (0.45–0.52)	0.09 (0.08–0.10)
75–79 years	0.26 (0.24–0.28)	0.50 (0.46–0.54)	0.08 (0.07–0.09)

### 3.3. Variation with period and cohort

**Figure 5:**
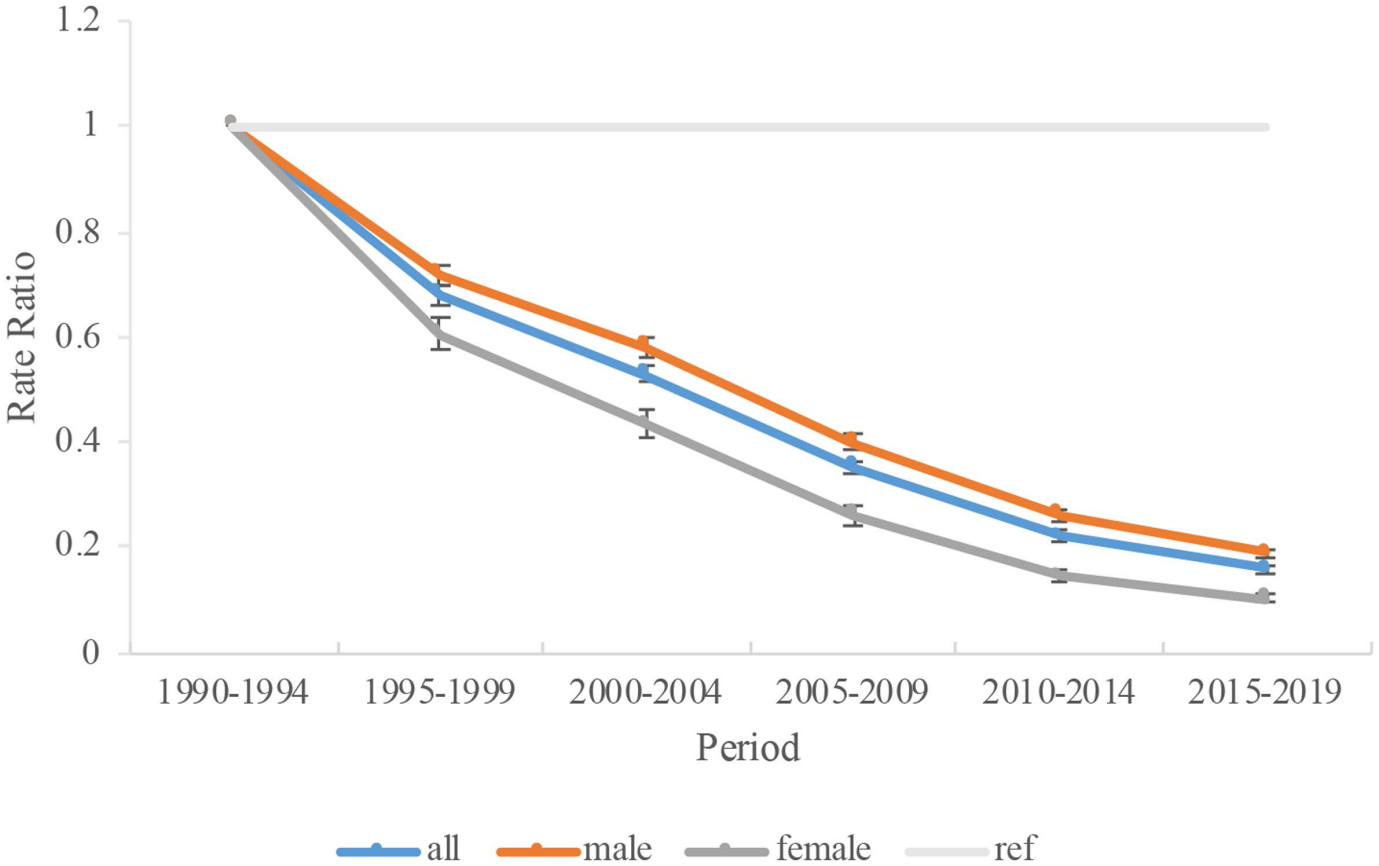
Period rate ratio (RR) of high fasting plasma glucose-attributable tuberculosis mortality in China. Data provided for all, male, and female. The RR of each period compared with the reference period (years 1990–1994) adjusted for age and nonlinear cohort effects. Error bars represent the 95% CIs for the period RR.

**Figure 6:**
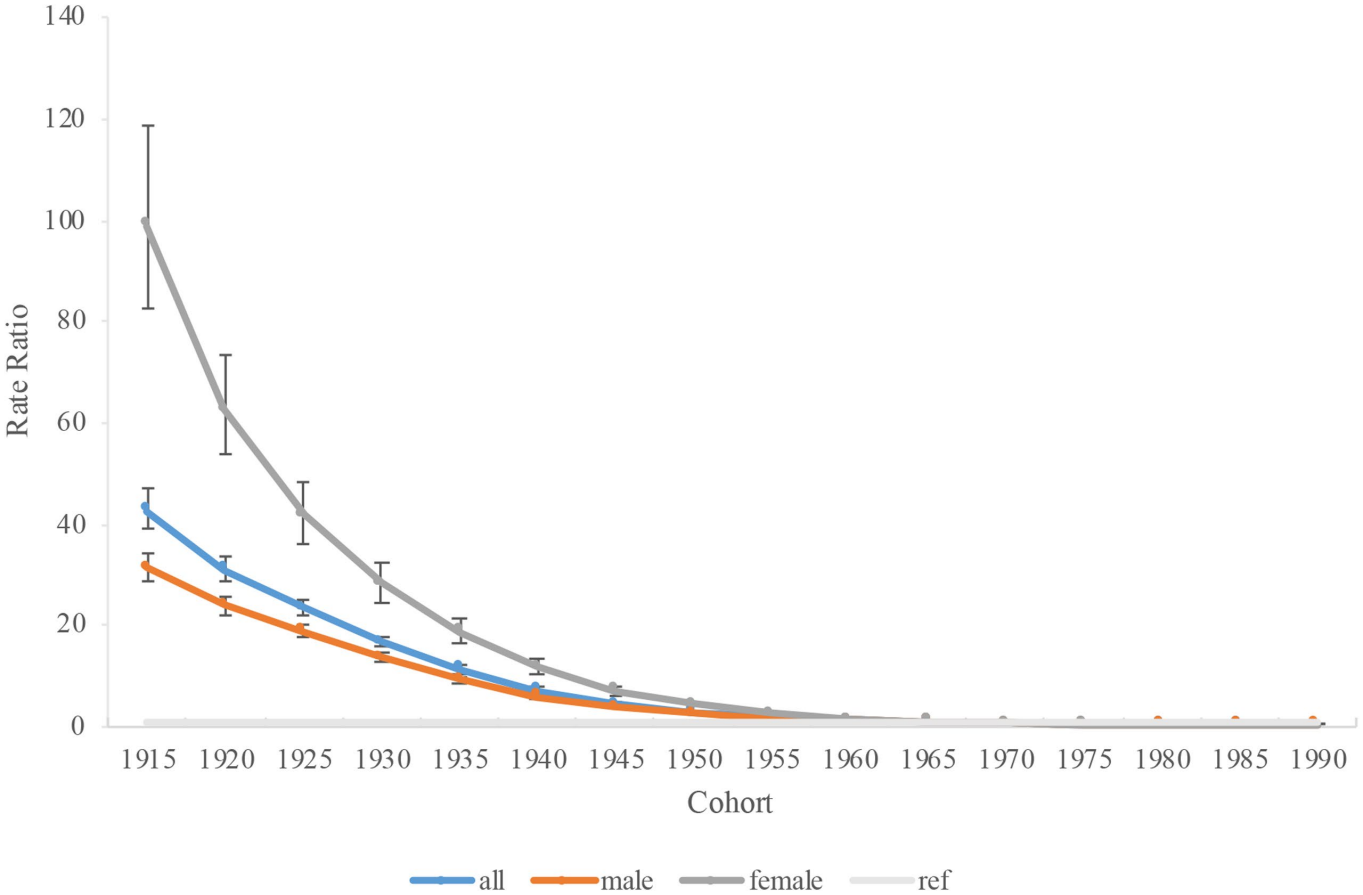
Cohort rate ratio (RR) of high fasting plasma glucose-attributable tuberculosis mortality in China. Data provided for all, male, and female. The RR of each cohort compared with the reference cohort (cohort 1965) adjusted for age and nonlinear period effects. Error bars represent the 95% CIs for the cohort RR.

The estimated period and cohort effects of HFPG-attributable TB mortality in China were depicted (Table 3). Figure 5 indicated a general decline in the risk of TB-related deaths attributable to HFPG from 1990 to 2019. Compared with the period of 1990–1994, the period RRs for TB has been consistently declining every period, which is 0.68 (0.66–0.70) in 1995 to 1999 and reach to 0.16 (0.15–0.16) in 2015–2019. Between 1990 and 2019, there were comparable trends observed in both men and women. Specifically, the mortality for men decreased from 0.72 (0.70–0.74) in 1995–1999 to 0.19 (0.18–0.20) in 2015–2019. For women, the rate decreased from 0.61 (0.57–0.64) in 1995–1999 to 0.10 (0.09–0.11) in 2015–2019. Compared with the cohort of 1965, the cohort RRs for TB were higher before 1965 but lower after 1965 (Figure 6). The data suggested that the decline in cohort effect was more prominent among women than among men.

**Table 3:** Sex-specific high fasting plasma glucose-attributable tuberculosis mortality in China due to period and cohort effects.

Factor	Mortality in all, RR (95%CI)	Mortality in male, RR (95%CI)	Mortality in female, RR (95%CI)
Period			
1990–1994	Ref	Ref	Ref
1995–1999	0.68 (0.66–0.70)	0.72 (0.70–0.74)	0.61 (0.57–0.64)
2000–2004	0.53 (0.51–0.55)	0.58 (0.56–0.60)	0.43 (0.40–0.46)
2005–2009	0.35 (0.34–0.37)	0.40 (0.38–0.42)	0.26 (0.24–0.28)
2010–2014	0.22 (0.21–0.23)	0.26 (0.25–0.27)	0.14 (0.13–0.16)
2015–2019	0.16 (0.15–0.16)	0.19 (0.18–0.20)	0.10 (0.09–0.11)
Cohort			
1915	42.82 (38.95–47.08)	31.35 (28.45–34.54)	99.12 (82.78–118.69)
1920	30.96 (28.58–33.54)	23.97 (22.12–25.96)	62.71 (53.57–73.42)
1925	23.4 (21.75–25.18)	18.82 (17.50–20.23)	41.88 (36.18–48.48)
1930	16.77 (15.65–17.97)	13.62 (12.72–14.59)	28.28 (24.6–32.51)
1935	11.38 (10.64–12.16)	9.25 (8.66–9.89)	18.87 (16.48–21.61)
1940	7.31 (6.85–7.81)	5.99 (5.62–6.40)	11.87 (10.39–13.57)
1945	4.57 (4.28–4.87)	3.82 (3.58–4.07)	7.22 (6.33–8.23)
1950	3.03 (2.84–3.22)	2.6 (2.45–2.77)	4.53 (3.99–5.14)
1955	1.98 (1.86–2.10)	1.79 (1.69–1.91)	2.57 (2.26–2.92)
1960	1.33 (1.24–1.42)	1.27 (1.19–1.36)	1.49 (1.30–1.70)
1965	Ref	Ref	Ref
1970	0.74 (0.68–0.80)	0.76 (0.70–0.82)	0.69 (0.59–0.80)
1975	0.53 (0.47–0.59)	0.57 (0.51–0.63)	0.43 (0.35–0.53)
1980	0.40 (0.34–0.46)	0.46 (0.40–0.54)	0.28 (0.20–0.38)
1985	0.31 (0.25–0.38)	0.39 (0.31–0.48)	0.19 (0.12–0.29)
1990	0.26 (0.18–0.37)	0.36 (0.25–0.52)	0.13 (0.06–0.26)

RR, rate ratio; CI, confidence interval.

## 4. Discussion

According to the current research, China’s ASMRs for TB related to HFPG were lower than the global average value. There was a significant decrease in ASMRs, period, and cohort RRs for TB attributable to HFPG over the period of 1990–2019, which was observed in both men and women. Even though men had higher mortality for TB, they experienced a smaller reduction in mortality compared to women. Moreover, The HFPG–related mortality of TB first increased and then decreased with the increase of age.

Nowadays, HFPG is regarded as the third highest risk factor for disease burden worldwide (21, 35). As the primary manifestation associated with HFPG, diabetes has posed a significant burden on society in terms of mortality, morbidity, and economic cost (7, 9). Due to the intricate pathophysiological effects of diabetes, the association between diabetes and TB cannot be attributed to a single mechanism. Several hypotheses have been proposed to explain their relationship, such as depressed cellular immunity, low levels of interferon-γ (IFN-γ), dysfunction of alveolar macrophages and pulmonary microangiopathy (36). The global diabetes epidemic poses a challenge to achieving Global End TB Targets. Evidence suggests that individuals with active TB and elevated blood glucose levels are at risk of experiencing negative outcomes, such as increased bacterial load in sputum and leucocytosis (37, 38). It is important to manage diabetes and maintain optimal glucose levels to reduce the likelihood for developing TB and significantly enhancing treatment effectiveness (39). The most significant decreasing in AAPC of HFPG-attributable TB mortality is in the 60–64 age group. This indicates that this particular age range is the critical period in a person’s life in terms of HFPG’s potential role in causing TB mortality. Therefore, interventions focused on managing diabetes and hyperglycemia during this stage of life would have the much significant impact on improving health outcomes for TB.

The Western Pacific Region is one of the six regions of the World Health Organization (WHO), which has been reported that the TB epidemic is more prevalent among the older adult (40, 41). In older adults, TB cases are frequently caused by the reactivation of dormant TB lesions. This reactivation is associated with changes in the immune system that occur during aging. For example, a reduction in the capacity to revive immunity that was acquired in the past (41, 42). While the TB mortality are still highest among older adult patients, particularly those aged 70 and above, there has been a decrease in mortality rates since 1990. Nevertheless, we observed the HFPG-attributable TB mortality first increases and then decreases with the increase of age, and peaked at 55–59 age group. Although advancing age is the main risk factor of TB death, high risk of death related to TB in the older adult might partly due to increased co-morbidities. Apart from diabetes, the contribution of HIV, drug use and chronic obstructive pulmonary disease is also important (43, 44). The GBD study highlights smoking, drinking, and HFPG as three risk factors associated with TB. However, ambient air pollution and other specific factors also could elevate the chances of the transmission and development of active TB. Notably, ambient air pollution is a significant public health issue and a primary contributor to the burden of disease. It is crucial to conduct a thorough assessment of the available data to understand the role of these risk factors in TB in future (45).

Our study showed that men had higher HFPG–related mortality of TB than women. Global data on morbidity and mortality indicate that men face a greater burden of TB than women, which is consistent with broader health patterns (46–48). On the other hand, nationally representative surveys conducted in China have shown that women exhibit lower prevalence of diabetes and prediabetes compared to men (11, 49). A recently study estimated the disease burden attributable to metabolic risk factors. It revealed that male patients with HFPG had a higher mortality than female patients in China (50). The elevated mortality in men may be attributed to variations in biological factors compared to women. For example, the impact of sex hormones on macrophage activation appears to differ by gender. Research has demonstrated that oestradiol, a female sex hormone, can significantly enhance the activation of macrophages. In contrast, androgen does not have the same effect (51, 52). Prior researches suggested that men who have lower levels of sex hormone-binding globulin are more susceptible to myocardial infarction, with free testosterone levels potentially playing a role in the development of major adverse cardiovascular events (53). It’s important to mention that testosterone has a significant impact on the susceptibility to TB based on sex (54–57). Results from an animal study revealed that orchidectomy performed after TB infection reduced the amount of bacteria and raised the level of IFN-γ and tumor necrosis factor alpha (TNF-α) (54). Moreover, testosterone levels might associate with diabetes status. Yoo et al. (57) indicated that the interplay between testosterone levels, diabetes status, and TB risk could result in men who have had diabetes for a longer duration being at the highest risk for TB. Additionally, mortality related to TB is linked to harmful health habits and risks can be significantly impacted by gender-specific norms and behaviors, which may vary between men and women. Men often exhibit delayed healthcare seeking behavior and may experience poorer treatment adherence compared to women (58, 59). In fact, a crucial obstacle in effectively managing glucose levels in TB patients is adhering to medication (60, 61). Hence, strategies for controlling HFPG should ought to be focused more on males.

The present study is of importance to public health as it provides a comprehensive analysis of the mortality trend of TB caused by HFPG in China during the years 1990–2019. In the past three decades, China has made remarkable progress in reducing both the occurrence and death rates of TB. Nonetheless, according to current TB control efforts in China, it is anticipated that the number of TB deaths will decrease to 26,038 in 2025, and 20,138 in 2030 (62). The number of TB-related deaths will remain considerably above the milestones and objectives established by the End TB Strategy (6, 62). In order to further decrease the burden of TB, it is essential to consistently enhance the prevention, diagnosis, and treatment of hyperglycemia in China, following the guidelines provided by the WHO. Moreover, public health policies can focus on strengthening healthcare infrastructure, ensuring an adequate healthcare workforce, and improving access to essential medicines, diagnostics, and treatments for both TB and HFPG. In future studies, it is important to evaluate the effectiveness of different strategies aimed at addressing the co-occurrence of TB and HFPG. By reducing the prevalence of HFPG and improving blood glucose control in HFPG patients, it helps to lower the risk of death from TB in individuals.

This study still has some limitations. Firstly, it did not incorporate information related to the data stratified by province and by urban and rural areas, which limited the ability to reveal more detailed information that influences TB mortality trends in China. Secondly, while the findings were derived from the GBD database, it was not possible to fully distinguish the independent impacts of HFPG, as it is always co-occurring with other risk factors including medication use, comorbidities like HIV/AIDS, socioeconomic status and risk factor management. Finally, estimation of the burden of TB mortality based on fasting glucose might underdiagnosis of the burden from subjects with postprandial hyperglycemia.

## 5. Conclusion

To sum up, the study shows declining trends of ASMRs and the risk of TB mortality related to HFPG in China from 1990 to 2019. Although these findings underscore the progress being made in combatting TB, it still has a long way to go to achieve the WHO’s End TB Strategy targets by 2030. Effective strategies for glycemic control and management are crucial for preventing TB in China, especially among middle aged and older adults and men. These strategies could include lifestyle changes such as diet and physical activity, as well as medication and regular blood glucose monitoring. Future researches are required to update and complement these estimates, and further explore underlying mechanisms.

## Data availability statement

Publicly available datasets were analyzed in this study. This data can be found here: the datasets presented in this study can be found in online repositories. The names of the repository/repositories and accession number(s) can be found at: All the data may be available from the IHME website: https://vizhub.healthdata.org/gbd-results/.

## Ethics statement

The studies involving human participants were reviewed and approved by data released from the Global Health Data Exchange query did not require informed patient consent. This study used an anonymized publicly available dataset with no identifiable information about the survey participants. Written informed consent for participation was not required for this study in accordance with the national legislation and the institutional requirements.

## Author contributions

CW wrote the manuscript. CW, XY, and HZ conducted the statistical analyses and interpreted the data. XY, HZ, CAW, YZ, JT, and XJ contributed to the manuscript revision, review for important intellectual content, and final version approval. CW and CAW conceived and designed this study. All authors contributed to the article and approved the submitted version.

## Funding

This work was supported by the Beijing Municipal Health Commission (BJRITO-RDP-2023), Beijing Talents Fund (2015000021469G178), and Beijing Jishuitan Hospital Nova Program (XKXX201801).

## Conflict of interest

The authors declare that the research was conducted in the absence of any commercial or financial relationships that could be construed as a potential conflict of interest.

## Publisher’s note

All claims expressed in this article are solely those of the authors and do not necessarily represent those of their affiliated organizations, or those of the publisher, the editors and the reviewers. Any product that may be evaluated in this article, or claim that may be made by its manufacturer, is not guaranteed or endorsed by the publisher.

## Supplementary material

The Supplementary material for this article can be found online at: https://www.frontiersin.org/articles/10.3389/fpubh.2023.1225931/full#supplementary-material
