# Supplementary material for: Temporal trends in mortality of tuberculosis attributable to high fasting plasma glucose in China from 1990 to 2019: a joinpoint regression and age-period-cohort analysis
Source: Front Public Health. 2023 Jul 27;11:1225931. doi: 10.3389/fpubh.2023.1225931 (PMC10413982; doi:10.3389/fpubh.2023.1225931)
Supplement: Supplementary file 1 [file Table_1.docx]

# Supplementary Tables

Table S1 Age-standardized mortality rates (per 100,000) of tuberculosis attributable to high fasting plasma glucose from 1990 to 2019 in China and globally.

| Year | Globally | | | China | | |
| --- | --- | --- | --- | --- | --- | --- |
|  | all | male | female | all | male | female |
| 1990 | 2.7 | 3.9 | 1.7 | 1.5 | 2.1 | 1.1 |
| 1991 | 2.7 | 3.9 | 1.7 | 1.5 | 2.1 | 1.0 |
| 1992 | 2.7 | 4.0 | 1.7 | 1.4 | 2.0 | 1.0 |
| 1993 | 2.7 | 3.9 | 1.7 | 1.4 | 1.9 | 0.9 |
| 1994 | 2.6 | 3.8 | 1.6 | 1.3 | 1.8 | 0.8 |
| 1995 | 2.5 | 3.7 | 1.6 | 1.2 | 1.7 | 0.7 |
| 1996 | 2.5 | 3.6 | 1.5 | 1.1 | 1.6 | 0.7 |
| 1997 | 2.5 | 3.6 | 1.5 | 1.0 | 1.5 | 0.6 |
| 1998 | 2.5 | 3.6 | 1.5 | 0.9 | 1.4 | 0.5 |
| 1999 | 2.4 | 3.6 | 1.5 | 0.9 | 1.3 | 0.5 |
| 2000 | 2.4 | 3.5 | 1.5 | 0.8 | 1.3 | 0.5 |
| 2001 | 2.3 | 3.4 | 1.4 | 0.8 | 1.2 | 0.5 |
| 2002 | 2.3 | 3.3 | 1.4 | 0.8 | 1.2 | 0.4 |
| 2003 | 2.2 | 3.2 | 1.4 | 0.8 | 1.2 | 0.4 |
| 2004 | 2.1 | 3.1 | 1.3 | 0.7 | 1.1 | 0.4 |
| 2005 | 2.0 | 3.0 | 1.3 | 0.7 | 1.0 | 0.4 |
| 2006 | 2.0 | 2.9 | 1.2 | 0.6 | 0.9 | 0.3 |
| 2007 | 1.9 | 2.8 | 1.2 | 0.5 | 0.8 | 0.3 |
| 2008 | 1.9 | 2.7 | 1.1 | 0.4 | 0.7 | 0.2 |
| 2009 | 1.8 | 2.6 | 1.1 | 0.4 | 0.6 | 0.2 |
| 2010 | 1.7 | 2.5 | 1.0 | 0.4 | 0.6 | 0.2 |
| 2011 | 1.7 | 2.4 | 1.0 | 0.3 | 0.5 | 0.2 |
| 2012 | 1.6 | 2.4 | 1.0 | 0.3 | 0.5 | 0.2 |
| 2013 | 1.6 | 2.4 | 1.0 | 0.3 | 0.5 | 0.1 |
| 2014 | 1.6 | 2.3 | 1.0 | 0.3 | 0.4 | 0.1 |
| 2015 | 1.6 | 2.2 | 1.0 | 0.3 | 0.4 | 0.1 |
| 2016 | 1.5 | 2.2 | 0.9 | 0.2 | 0.4 | 0.1 |
| 2017 | 1.5 | 2.1 | 0.9 | 0.2 | 0.3 | 0.1 |
| 2018 | 1.5 | 2.1 | 0.9 | 0.2 | 0.3 | 0.1 |
| 2019 | 1.5 | 2.1 | 0.9 | 0.2 | 0.3 | 0.1 |
